# Strengthening HIV and HIV co‐morbidity care in low‐ and middle‐income countries: insights from behavioural economics to improve healthcare worker behaviour

**DOI:** 10.1002/jia2.26074

**Published:** 2023-04-03

**Authors:** Anant Mishra, Tonderai Mabuto, Kate Shearer, Antonio Trujillo, Jonathan E. Golub, Christopher J. Hoffmann

**Affiliations:** ^1^ Department of Medicine Johns Hopkins University School of Medicine Baltimore Maryland USA; ^2^ Implementation Research Division The Aurum Institute Johannesburg South Africa; ^3^ The University of the Witwatersrand School of Public Health Johannesburg South Africa; ^4^ Department of International Health Johns Hopkins Bloomberg School of Public Health Baltimore Maryland USA; ^5^ Department of Epidemiology Johns Hopkins Bloomberg School of Public Health Baltimore Maryland USA; ^6^ Department of Health, Behavior, and Society Johns Hopkins Bloomberg School of Public Health Baltimore Maryland USA

**Keywords:** behavioural economics, care delivery, healthcare worker behaviour, HIV/AIDS, implementation science, LMICs

## Abstract

**Introduction:**

Despite advances in HIV and HIV co‐morbidity service delivery, substantial challenges remain in translating evidence‐based interventions into routine practice to bring optimal care and prevention to all populations. While barriers to successful implementation are often multifactorial, healthcare worker behaviour is critical for on‐the‐ground and in‐clinic service delivery. Implementation science offers a systematic approach to understanding service delivery, including approaches to overcoming delivery gaps. Behavioural economics is a field that seeks to understand when and how behaviour deviates from traditional models of decision‐making, deviations which are described as biases. Clinical policies and implementation strategies that incorporate an understanding of behavioural economics can add to implementation science approaches and play an important role in bridging the gap between healthcare worker knowledge and service delivery.

**Discussion:**

In HIV care in low‐ and middle‐income countries (LMICs), potential behavioural economic strategies that may be utilized alone or in conjunction with more traditional approaches include using choice architecture to exploit status quo bias and reduce the effects of cognitive load, overcoming the impact of anchoring and availability bias through tailored clinical training and clinical mentoring, reducing the effects of present bias by changing the cost–benefit calculus of interventions with few short‐term benefits and leveraging social norms through peer comparison. As with any implementation strategy, understanding the local context and catalysts of behaviour is crucial for success.

**Conclusions:**

As the focus of HIV care shifts beyond the goal of initiating patients on antiretroviral therapy to a more general retention in high‐quality care to support longevity and quality of life, there is an increasing need for innovation to achieve improved care delivery and management. Clinical policies and implementation strategies that incorporate elements of behavioural economic theory, alongside local testing and adaptation, may increase the delivery of evidence‐based interventions and improve health outcomes for people living with HIV in LMIC settings.

## 1 INTRODUCTION

Advances in HIV management, delivery models and accelerated antiretroviral therapy (ART) initiation have markedly reduced HIV mortality, morbidity and transmission [1]. However, gaps in the delivery of these services limit the full potential of these advances, including in low‐ and middle‐income countries (LMICs). While factors such as a lack of resources contribute to delivery challenges, in many cases, gaps in care persist despite available medications, diagnostics and guidelines [2, 3, 4, 5]. For example, despite World Health Organization recommendations, less than half of eligible people living with HIV are initiated on tuberculosis preventive therapy (TPT), and even fewer receive recommended hypertension management [5, 6]. Barriers to the implementation of these and other interventions occur at several levels, but direct care delivery by frontline healthcare workers continues to play an appreciable role [7, 8, 9]. Devising strategies that address healthcare worker behaviour can improve patient care and outcomes in LMICs.

Implementation science is a field focused on developing and promoting the sustainable adoption and integration of evidence‐based practices and policies into routine healthcare and public health settings [10]. Implementation science theories often include healthcare worker behaviour, alongside their relationship and attitude towards an intervention, as important determinants of implementation [11, 12, 13, 14]. These theories suggest that the presence of facilitating determinants, such as healthcare worker motivation and training, increases the potential for effective intervention delivery. However, there are important instances when interventions are incompletely implemented for reasons not described by traditional implementation considerations. An illustrative example from a high‐income setting is the variation in services a healthcare worker provides over the course of the day to a similar patient population [15]. In this example, all determinants of implementation remain identical—healthcare worker knowledge, resources and motivation—yet healthcare workers are less likely to provide vaccination to the same patient population later in the clinical day. This behaviour, and other modulators of implementation, are not completely explained by a fully deductive or rational evaluation of implementation.

Behavioural economics is a field that draws on learnings from social psychology and economic theory and offers insights that can inform implementation strategies. In particular, implementation theories tend to utilize more linear reasoning grounded in rational choice theory. Rational choice theory predicts that an individual with specific knowledge, preferences and relevant information will make decisions that are predictable based on maximizing long‐term satisfaction or utility [16]. In the vaccination and time of day example, the healthcare worker's preferences and available information are not expected to change during the day, but the decision to recommend vaccination does. Behavioural economics provides an approach to understanding this and other behaviour that does not fully conform to the predictions of rational choice theory [16]. Behavioural economics posits that behaviour that deviates from the expectations of rational choice theory tends to do so with predictable patterns [17]. These patterns are often described as biases [17]. By understanding the nature of these biases and the context in which they occur, strategies can be employed to increase desired healthcare worker behaviour and improve intervention delivery.

Here, we seek to illustrate applications for incorporating insights from behavioural economics into implementation strategies focused on healthcare worker behaviour for HIV and integrated co‐morbidity service delivery in LMICs. We approach this topic through exploring four commonly considered biases (of many described biases): status quo bias and cognitive load, anchoring and availability bias, present bias and social norms (Table 1). These biases were selected as examples based on the review of the literature, relevance to HIV and HIV co‐morbidity care, and experience of the authors in implementation research, policy planning and clinical delivery in LMICs. Other biases may also be relevant to specific service delivery questions and can be further explored using available resources [18, 19]. It is also important to note that some biases are referred to by several terms but share common underlying principles, and may be used interchangeably here (e.g. availability bias and salience). Finally, although there is a place for behavioural economics to improve care delivery in all settings, our focus is centred on HIV and related co‐morbidity care in LMICs given the high burden of HIV in these regions, opportunities provided by the commonly used public health approach to HIV care and the urgent need for innovative implementation strategies.

**Table 1:** Overview of cognitive biases and behavioural economic strategies to improve HIV and HIV co‐morbidity care in low‐ and‐middle‐income countries (LMICs)

Bias	Role in health delivery	Impact on HIV care	Policies and implementation strategies
Status quo bias and cognitive load	Status quo bias refers to the tendency for individuals to maintain the current state or default [20]. The tendency to accept the status quo increases as cognitive load grows [21]. This may lead healthcare workers to maintain the status quo and avoid altering the current testing or treatment plan even if suboptimal.	Healthcare workers face high cognitive load and increasingly accept the status quo as a result, which can lead to suboptimal care [15, 22, 23, 24, 25, 26].	Simplified care algorithms or checklists may decrease cognitive load and increase delivery. Choice architecture approaches can also improve intervention delivery. Examples in HIV care include reflex serum cryptococcal antigen screening and routine same day ART initiation [27, 28].
Anchoring and availability bias	Anchoring bias may cause clinicians to hold on to an initial clinical assessment and fail to reconsider alternative diagnoses as new evidence is introduced [36]. Availability bias refers to the tendency to judge the probability of events based on how readily it comes to mind. Because dramatic examples tend to be most readily recalled, healthcare workers may misjudge the likelihood of dramatic events even if rare [36].	Anchoring bias may contribute to a clinician's reluctance to prescribe TB preventive therapy due to continued suspicion of TB disease even after new evidence provides an alternative diagnosis [31, 32, 33]. Availability bias may reduce TB preventive therapy prescribing if a clinician recalls memorable rare adverse reactions [32, 33].	Tailored clinical training and mentoring along with clear clinical management algorithms can help healthcare workers better estimate the outcomes of clinical decisions, identify biases affecting their clinical decision‐making and reflect further on strategies to mitigate additional individual biases [40].
Present bias	Present bias refers to the tendency of individuals to more heavily discount future benefits and costs [41]. Thus, healthcare worker effort directed to improve distant health outcomes may take a backseat to issues that appear to be of more immediate concern, even if delaying has large future costs.	Care to improve long‐term health, such as preventive care, including vaccinations, cancer screening and HIV testing, may be repeatedly postponed to the “next visit” [42, 43].	Simplifying procedures (e.g. pre‐filled medicine bottles) to reduce present costs may help improve the delivery. P4P programmes have increased ART initiations and coverage [47, 48].
Social norms	Clinic and healthcare worker behaviour is influenced by perceptions of how peers and colleagues perform due to a desire to conform to social norms, the informal social understandings that govern the behaviour of the members of a group or society [50, 51].	A healthcare worker may seek to provide a similar level of care as their peers in terms of HIV testing or viral load monitoring.	Social norm‐based strategies like peer comparison have been used to improve healthcare worker behaviour [54, 55, 56, 57, 58]. Facility‐level comparison has been used to improve ART initiation and viral load monitoring [59, 60].

Abbreviations: ART, antiretroviral therapy; HPV, human papillomavirus; LMICs, low‐ and middle‐income countries; P4P, pay for performance.

## 2 DISCUSSION

### 2.1 Status quo bias and cognitive load

Status quo bias or default bias refers to the tendency of individuals to prefer the current state of affairs—the status quo [20]. This is driven in part because deviating from the status quo requires an active decision which requires mental effort or cognitive load that could instead be conserved [20]. Consequently, in a busy healthcare environment where the cognitive load is high as a result of having to make numerous small and large decisions, healthcare workers may sometimes passively accept the default and not specifically make the decision to recommend an HIV test, not screen for a disease or not prescribe certain indicated medications. This tendency to accept the status quo is even more pronounced as the cognitive load of the action increases (i.e. it becomes more complex) or as an individual's cumulative cognitive load increases throughout the day [21]. This may help explain the previously mentioned decrease in vaccination rates (and other interventions) during the day as healthcare workers’ cognitive load accrues [15, 22, 23, 24]. It is clear that status quo bias can impede care delivery, especially when a healthcare worker faces high cognitive load like that commonly seen in healthcare settings in LMICs [25, 26].

Understanding the relationship between status quo bias and cognitive load can be used to improve service delivery. For example, simplifying care algorithms, employing checklists or setting the usually favoured clinical decision as the default choice (the action taken if no decision is made) are all approaches that can reduce cognitive load on the healthcare worker and increase delivery of the specified service [16].

This deliberate selection of the way choices are presented is referred to as choice architecture and is already being utilized within HIV and HIV co‐morbidity care in LMICs [16]. Policy changes that mirror choice architecture approaches (whether deliberate or otherwise) have been adopted for some practices, including default reflex serum cryptococcal antigen screening on CD4^+^ cell count test specimens if the CD4^+^ count result is <100 cells/μl (which increases the chance of appropriate testing and reduces the time to test results) and universal HIV treatment policies (which shifted the prior approach of only considering ART for selected patients to the “default” of ART initiation for all) [27, 28]. Another intentional choice architecture‐based implementation strategy is shifting TPT prescribing from a default of non‐prescribing unless strict eligibility criteria are met to default prescribing to all patients unless the healthcare worker identifies that TPT should not be prescribed and actively makes the choice not to prescribe TPT [clincaltrials.gov NCT04466488; clinicaltrials.gov NCT04466293]. This approach may overcome the high cognitive load associated with navigating the often multi‐step TPT prescribing algorithm, which has been identified as a barrier to TPT delivery [29, 30, 31, 32, 33]. A similar approach might be considered to improve service delivery when guideline complexity may lead to under‐implementation such as with viral load monitoring where guidelines are often multi‐faceted and complex [34, 35].

### 2.2 Anchoring and availability bias

Anchoring bias refers to the tendency for initial information or premature conclusions to outweigh subsequent and potentially contradictory information [36, 37]. Availability bias refers to the propensity to (mis)judge the likelihood of an event based on the prior experiences that most readily come to mind [36]. Because rare dramatic events—a highly unlikely clinical complication, a remarkable recovery, and so on—are often more readily recalled, these rare events can skew perceptions of the risk or benefit of a healthcare intervention [36, 37, 38, 39] (Table 1).

The effects of anchoring and availability bias on treatment decisions are wide‐ranging. Anchoring bias may contribute to a healthcare worker's reluctance to prescribe TPT to a patient if they remain anchored to an initial diagnosis of tuberculosis (TB) disease even after new evidence provides an alternative explanation of symptoms [31, 32, 33]. Availability bias may also reduce TPT prescribing if a healthcare worker overestimates the risk of a rare but severe adverse reaction, such as a drug‐induced liver injury, based on experience with a prior patient or an anecdote from a colleague [32, 33]. Approaches that may reduce the effect of these biases are tailored clinical training and clinical mentoring along with clear clinical management algorithms [40]. These approaches may help healthcare workers better estimate the outcomes of clinical decisions, identify biases affecting their decision‐making and reflect further on strategies to mitigate additional individual biases [40].

### 2.3 Present bias

Present bias refers to the tendency of individuals to excessively discount the value of future benefits and costs compared to those in the present [41]. That is, the current cost of an intervention, despite potential long‐term benefits, may result in delivery being neglected while more immediate issues are addressed, even if a delay may have considerable future health costs. This is most prominent in the delivery of preventive care, including vaccinations, cancer screening and HIV testing that may be repeatedly postponed to the “next visit” such as has been reported with human papillomavirus (HPV) vaccinations [42, 43]. The effects of present bias on intervention implementation may be reduced by decreasing the present cost to a healthcare worker or increasing a healthcare worker's perceived value of the long‐term benefits.

Reducing present costs in the clinical setting (time and effort required from the healthcare worker) may reduce present bias and improve delivery. Interventions that involve simplifying procedures, such as having pre‐selected order sets, pre‐filled medicine bottles, readily available specimen bottles for sample collection, informational material for patients to review prior to seeing a healthcare worker or pre‐screening for vaccination by ancillary clinical staff, are all potential approaches.

Overcoming the effects of present bias may also involve increasing the present value of an action to a healthcare worker. One approach is to emphasize the future value gained from undertaking an action. This might be achieved by having healthcare workers care for patients with advanced, but preventable illnesses (such as HPV‐related cancers). Another approach is the use of financial incentives. Financial incentives, when used as part of a behavioural economics approach, need to be large enough to influence healthcare worker decisions, but not so large that they rise to the level of full payment for a service which more closely resembles the direct compensation approach of traditional economic theory [44]. Pay for performance (P4P) programmes that financially reward healthcare workers a small amount for meeting certain service targets are one example which have shown promise [45, 46]. A review of HIV care‐focused P4P schemes found improvements in ART coverage [47], while an analysis of a P4P programme in Mozambique found increases in ART initiations for certain vulnerable populations living with HIV [48]. Behavioural economics offers further insights to potentially strengthen the efficacy of these programmes [49]. P4P programmes can increase the salience of the incentives to healthcare workers by reducing the time between desired action and incentive payout and also clearly distinguishing performance incentives from base salary. Similarly, programmes might draw on the idea of loss aversion—the idea that individuals respond more readily to a loss than a gain—by structuring incentives such that they are disbursed at the beginning of the pay period but must be returned if goals are not achieved. As with any implementation strategy, potential negative consequences need to be considered during the design process.

### 2.4 Social norms

Healthcare worker behaviour, like that behaviour of most individuals, is influenced by perceptions of how peers and colleagues perform [50]. For example, a healthcare worker may seek to provide a similar level of care as peers in order to conform to social norms—the informal social understandings that govern the behaviour of members of a group or society [51].

One social norm‐based approach to improve delivery is to compare healthcare worker performance metrics with peers in order to change behaviour [52, 53]. Studies of peer comparison from high‐income settings focused on bolstering HIV and HCV screening rates, improving cardiovascular disease management for people living with HIV and strengthening the overall quality of patient care have demonstrated positive and sustained improvements in behaviour [54, 55, 56]. Two studies that utilized peer comparison to improve TB care in LMICs demonstrated increases in guideline‐adherent screening and care for TB patients [57, 58]. At the facility level, comparisons between clinics have also been studied as a strategy for improving service delivery, including as a multicomponent intervention that successfully increased rapid ART initiation and viral load monitoring (Table 1) [59, 60]. Importantly, while the cited studies have highlighted the promise of peer comparison strategies, implementation is not without context‐dependent challenges that should be assessed. For example, peer comparison in settings where poor performance is widespread may be difficult to implement and could reinforce the non‐desired status quo.

### 2.5 Implementation considerations and limitations

Understanding the local context and catalysts of behaviour is crucial for the success of any implementation strategy, including those drawing on behavioural economic principles. Only after the determinants of implementation are understood can strategies be rationally designed for the local context. Through this evaluation, specific strategies can be developed based in behavioural economics. Existing behavioural economic frameworks can serve as resources for this process. For example, the MINDSPACE framework (Messenger, Incentive, Norms, Defaults, Salience, Priming, Affects, Commitments, Ego) outlines a list of nine important influences on behaviour that can be explored during intervention design [61]. As part of any implementation strategy, including those based on behavioural economics, ethical implications, potential unintended negative consequences and equity of care delivery need to be understood and appropriately managed [62].

While the underlying principles of behavioural economics can be expected to be universally applicable, approaches drawing on those principles require adaptation and empiric testing in the target setting. This is especially relevant when seeking to adapt behavioural economic‐based strategies to LMICs as the majority of their use has been in high‐income countries. Increased design, experimentation and evidence generation is needed to develop implementation strategies that effectively understand and utilize cognitive biases in varied LMIC settings. Notably, a particular strength of behavioural economic strategies is that they can be adapted to a wide range of environments and contexts.

## 3 CONCLUSIONS

Behavioural economics provides a powerful theoretical basis for policy and implementation strategy design and development. As the focus of HIV care shifts beyond the goal of initiating patients on ART to ensuring access to and retention in high‐quality care to support healthy longevity and quality of life, there is an increasing need for innovation to achieve improved delivery of the care continuum, treatment monitoring, preventive care and co‐morbidity management. Through a careful understanding of the effect of cognitive biases, insights from behavioural economics can be utilized to support novel and sustainable improvements in HIV and HIV co‐morbidity service delivery.

## COMPETING INTERESTS

The authors have nothing to disclose.

## AUTHORS’ CONTRIBUTIONS

AM and CJH made substantial contributions to the conceptualization of the work. All authors made contributions to the drafting of the work and all authors read and approved the manuscript's final version.

## Data Availability

The research reported in this publication was supported by R01AI150432 (CJH) from the National Institutes of Health (National Institute of Allergy and Infectious Diseases).
